# Adaptive graph-based multiple testing procedures

**DOI:** 10.1002/pst.1640

**Published:** 2014-10-16

**Authors:** Florian Klinglmueller, Martin Posch, Franz Koenig

**Affiliations:** Center for Medical Statistics, Informatics, and Intelligent Systems, Medical University of Vienna, Spitalgasse 23, 1090 Vienna, Austria

**Keywords:** multiple comparisons, treatment selection, multiple endpoints, partial conditional error rate, adaptive design, graphical approach

## Abstract

Multiple testing procedures defined by directed, weighted graphs have recently been proposed as an intuitive visual tool for constructing multiple testing strategies that reflect the often complex contextual relations between hypotheses in clinical trials. Many well-known sequentially rejective tests, such as (parallel) gatekeeping tests or hierarchical testing procedures are special cases of the graph based tests. We generalize these graph-based multiple testing procedures to adaptive trial designs with an interim analysis. These designs permit mid-trial design modifications based on unblinded interim data as well as external information, while providing strong family wise error rate control. To maintain the familywise error rate, it is not required to prespecify the adaption rule in detail. Because the adaptive test does not require knowledge of the multivariate distribution of test statistics, it is applicable in a wide range of scenarios including trials with multiple treatment comparisons, endpoints or subgroups, or combinations thereof. Examples of adaptations are dropping of treatment arms, selection of subpopulations, and sample size reassessment. If, in the interim analysis, it is decided to continue the trial as planned, the adaptive test reduces to the originally planned multiple testing procedure. Only if adaptations are actually implemented, an adjusted test needs to be applied. The procedure is illustrated with a case study and its operating characteristics are investigated by simulations.

## 1. INTRODUCTION

Clinical trials often address several study objectives within a single confirmatory experiment, and multiple hypothesis tests are part of the confirmatory statistical analysis. For example, non-inferiority and superiority hypotheses [1,2], several doses or treatment regimens, multiple endpoints [3], or multiple (sub-)populations can be investigated simultaneously in one clinical trial. To prevent inflated false positive rates due to multiple hypothesis testing, regulatory guidelines [4,5] require the control of the familywise error rate (FWER) in the strong sense. Accordingly, for a wide range of settings, specific multiple testing procedures have been developed [6]. In particular, testing strategies have been proposed that map the difference in importance and the logical relationships between hypotheses onto the multiple testing procedure. For example, in a clinical trial where high and low doses are compared with a control, the proof of superiority for the low dose may only be of interest if superiority for the high dose has been shown. More complex relations between hypotheses can occur if hypotheses corresponding to several treatment arms, endpoints, and subgroups are tested in a single experiment. O’Neill [7] notes, for example, that secondary endpoints shall not be tested before efficacy in the primary endpoint has been shown.

An intuitive tool to construct testing procedures that satisfy such requirements are directed, weighted graphs [8–10]. The graphs visually represent the testing strategy and implicitly define a sequentially rejective multiple testing procedure that controls the FWER. Many classical sequentially rejective tests, such as (parallel) gatekeeping tests [11,12], fixed sequence (‘hierarchical’) tests [13–15], or fall back procedures [16,17], are special cases of these graph-based tests. The graph-based tests belong to the general class of sequentially rejective weighted Bonferroni tests [18], which are based on the application of the closed testing principle [19] to weighted Bonferroni tests for intersection hypotheses.

In this manuscript, we extend the multiple testing procedures defined by weighted directed graphs to adaptive tests controlling the FWER in the strong sense. Boosted by the publication of regulatory guidance documents [20,21], adaptive designs have attracted much attention over the last decade. Although the most frequently studied type of adaptation is sample size reassessment [22–27], more substantial modifications have been considered in settings where multiple hypotheses are tested. Such adaptations include the selection of treatment arms, subgroups, or endpoints [28–38], see [39] for a review on confirmatory adaptive designs based on combination tests and conditional error functions. In a confirmatory setting, adaptive changes of the trial design based on unblinded interim data must not compromise the integrity of the trial and a minimal requirement is the control of the FWER.

The adaptive graph-based testing procedure proposed in this manuscript allows one to adapt the design of an ongoing trial for which a multiple hypothesis test has been prespecified using the graph-based approach. The procedure is applicable in a wide range of scenarios including trials with multiple treatment comparisons, endpoints, or subgroups and allows for the adaptation of sample sizes, selection of treatment arms, subgroups, or endpoints and the graph-based multiple testing strategy itself. The adaptations may be based on unblinded interim data as well as data from external sources, and the procedure controls the FWER in the strong sense without the need to prespecify the adaptation rule in detail.

The proposed adaptive test is based on a generalization of weighted Bonferroni intersection hypothesis tests to adaptive tests using partial conditional error rates. The procedure has the appealing property that in case no adaptations are performed at the interim analysis, the originally planned graph-based multiple testing procedure can be used. Only if the trial design is actually modified, the adaptive test needs to be applied. In contrast, adaptive multiple testing procedures based on combination tests [28–32,39] require test statistics based on combination functions of stagewise multiplicity adjusted test statistics, even if no adaptations are performed. Furthermore, the proposed adaptive testing procedure uniformly improves a recently suggested adaptive graph-based partitioning test procedure based on combination tests of stagewise elementary test statistics [40].

The manuscript is organized as follows. In Section 2, we review sequentially rejective weighted Bonferroni tests and their construction via directed weighted graphs. In Section 3, these tests are generalized to adaptive tests. First, in Section 3.1, partial conditional error rates [41,42] are used to derive conditional—on observations from subjects recruited in the first stage—significance levels of general weighted Bonferroni tests. Then, in Section 3.2, we construct corresponding weighted adapted second stage tests. In Section 4, we illustrate the approach with a case study in the spirit of the multi-armed multiple sclerosis trial considered in [8], where a treatment arm is dropped in an interim analysis and the sample size of the dropped arm is re-allocated to the remaining arms. For the scenario of this case study, we investigate the operating characteristics of the adapted test with simulations in Section 5. Finally, in Section 6, we discuss limitations and potential generalizations.

## 2. GRAPH-BASED MULTIPLE TESTING PROCEDURES

In this section we review fixed sample (non-adaptive) graph-based multiple test procedures that will be generalized to adaptive tests in Section 3. Consider the problem of testing *m* elementary null hypotheses *H_i_*, *i* ϵ *I* = {1, … *m*} controlling the FWER in the strong sense at level *α* such that the probability of at least one erroneous rejection is bounded by *α* under any configuration of true and false null hypotheses *H_i_*, *i* ϵ *I*.

**Figure 1:**
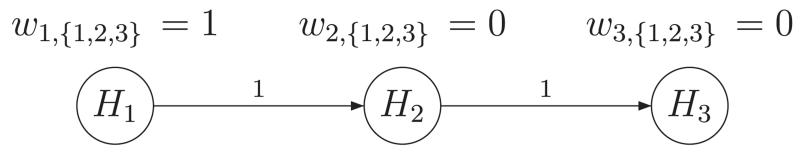
Graphical weighting procedure resulting in a hierarchical test of three elementary null hypotheses *H*_1_, *H*_2_, and *H*_3_.

Multiple testing based on graphs formalizes the following heuristic approach. Initially, the *m* hypotheses are tested, each at their local significance level *α_i_* = *w_i_*_,_*_I_α*, where the *w_i_*_,_*_I_* are weights, with 0 ⩽ *w_i_*_,_*_I_* and Σ_*i*ϵ*I*_ *w*_*i,I*_ ⩽ 1, that determine the initial allocation (i.e. for the global intersection hypothesis *H_I_* : ∩_*iϵI*_ *H_i_*) of the overall significance level across hypotheses. If a hypothesis *H_i_* can be rejected, its level is reallocated to the remaining hypotheses according to a prespecified rule. The testing step is then repeated for the remaining, non-rejected hypotheses with the updated local significance level. If a further null hypothesis can be rejected, its local significance level is reallocated using an updated allocation rule. This procedure is repeated until no further hypothesis can be rejected. This heuristic approach can be easily described by weighted, directed graphs, where the nodes correspond to hypotheses and the weights of directed edges determine the fraction of the local level that is reallocated to each of the other nodes after a hypothesis has been rejected. For example, a hierarchical test of three hypotheses is defined by the graph in Figure 1. Bretz *et al.* [8] have shown that (after a suitable formalization) the graphs define a multiple testing procedure that controls the FWER in the strong sense at level *α*. For a related graph-based approach, see [9].

To generalize the graph-based test to an adaptive test, we use the fact that the former is a sequentially rejective weighted Bonferroni test [8,43], which in turn is a shortcut of the closed testing procedure applied to weighted Bonferroni tests for all intersection hypotheses [18]. To define this closed testing procedure, one needs to consider all non-empty subsets *J* of *I* and specify non-negative weights, ***w**_J_* = (*w*_1,*J*_, …, *w_m,J_*), with *w_i_*_,__*J*_ = 0 for all *i* ∉ *J* and Σ_*j*ϵ*J*_ *w*_*j,J*_ ⩽ 1 (hereafter, we write *J, J* ⊆ *I* to denote all non-empty subsets of *I*). Also, let ***p*** = (*p*_1_, …, *p_m_*) denote the marginal unadjusted *p*-values. The corresponding weighted Bonferroni test rejects intersection hypothesis *H_J_* = ⋂_*j*ϵ*J*_ *H_j_* if any of the unadjusted *p*-values *p_j_*, *j* ϵ *J* falls below the weighted critical boundary *w*_*j,J*_*α*. This corresponds to a decision function *φ*_J_(***p***, *α*) = max_*j*ϵ*J*_ **1**_{*p_j_*⩽*w_j,J_α*}_ that takes the value of 1 if *H_J_* is rejected and zero otherwise. The closure test rejects an elementary hypothesis *H_i_*, *i* ϵ *I*, if *H_i_* and all intersection hypotheses *H_J_* = ⋂_*j*ϵ*J*_ *H_j_* with *J* ⊆ *I*, *i* ϵ *J* can be rejected each at (local) level *α*. This procedure corresponds to a decision function *ψ*_*i*_(***p***, *α*) = min_*J*⊆*I*, *i*ϵ*J*_ *φ*_*J*_(***p***, *α*) for each elementary hypothesis *H_i_* and controls the FWER at level *α* in the strong sense [19].

### 2.1. Defining weighted intersection hypothesis tests with graphs

Consider a weighted directed graph with *m* nodes where each node represents an elementary hypothesis *H_j_*, *j* ϵ *I*. For each of the nodes, we define a node weight and denote the corresponding vector of node weights by ***w***_*I*_ = (*w*_1,*I*_, …, *w*_*m,I*_). The nodes are connected by directed edges with edge weights *g*_*ij,I*_, 0 ⩽ *g*_*ij,I*_, Σ_*j*ϵ*I*_ *g*_*ij,I*_ ⩽ 1, and *g*_*ii,I*_ = 0 for all *i,j* ϵ *I*. Note that *g*_*ij,I*_ > 0 indicates a directed edge from *H_i_* to *H_j_*, *i,j* ϵ *I*, with positive weight. Let *G*_*I*_ = (*g*_*ij,I*_)_*i,j* ϵ *I*_ denote the *m* × *m* matrix of edge weights.

For the global null hypothesis *H_I_* : ⋂_*i*ϵ*I*_ *H*_*i*_, the node weights ***w**_I_* define a weighted Bonferroni test. To compute the weights for all intersection hypotheses *H_J_*, *J* ⊂ *I*, a stepwise algorithm specified by the edge weights *G_I_* is used (see Appendix A (available online as Supporting Information) for the technical details).

For example, to obtain the node weights ***w**_J_* for some *J* ⊂ *I*, first, compute the weights ***w***_*I*\{*ℓ*}_ for an arbitrary *ℓ* ϵ *I*\*J*. To this end, allocate the weight *w*_*ℓ,I*_ proportional to the edge weights *g*_*ℓj,I*_ (of edges *j* leaving the node *ℓ*) to the remaining hypotheses *H_j_*, *j* ϵ *I* \ {*ℓ*}. Now, remove node *ℓ* and all edges attached to it from the graph and update the remaining edge weights to obtain *G*_*I*\{*ℓ*}_. Repeat these steps (recursively allocating weights and updating the graph) for all further indices in *I* \ (*J* ∪ {*ℓ*}). The resulting weights are independent of the order in which the procedure is applied to the *ℓ* ϵ *I* \ *J* [8,43]. Because the graphical algorithm is uniquely specified by only *m* node weights and *m*^2^ – *m* edge weights, it covers only a subclass of all possible weighted-closed testing procedures.

The closure of the weighted Bonferroni intersection tests with weights defined by the aforementioned algorithm are equivalent to those of the corresponding graph-based sequentially rejective test that formalizes the heuristic approach to construct multiple tests discussed earlier. However, the formulation as a closed test allows one to generalize it to a multiple test procedure for adaptive study designs that controls the FWER in the strong sense. This is the topic of the next section.

## 3. ADAPTIVE WEIGHTED BONFERRONI TESTS

To derive adaptive weighted Bonferroni tests, we apply the partial conditional error approach [41,42,44] to weighted Bonferroni tests. The procedure is based on the conditional error rate methodology [45,46] that is based on the probability of a type I error of a preplanned test conditional on the data that have been observed up to the point of an unblinded interim analysis. To achieve strict type I error control, if the preplanned design is adapted (e.g., the sample size is modified), it is replaced by a test with conditional type I error rate below the conditional error rate of the preplanned test. Theoretically, adaptations can be based on internal or external data, and even the timing of the interim analysis does not have to be scheduled a priori in order to achieve strict control of the type I error rate.

For multiple hypothesis tests, the computation of the conditional error rate requires knowledge of the joint conditional (on the first stage observations) null distribution of the *p*-values corresponding to the investigated null hypotheses. Although in special cases, as many-to-one comparisons of normally distributed measurements, the conditional error rate can be computed directly [47], this approach fails if the correlation structure is unknown (for example, if multiple endpoints are tested). Therefore, we consider a test based on partial conditional error rates, which only requires that the marginal conditional null distributions are known at interim.

### 3.1. General adaptive weighted Bonferroni tests based on partial conditional error rates

We start out with a fixed sample closed test of weighted Bonferroni intersection hypothesis tests as defined in Section 2. Let *p_j_* denote the unadjusted marginal *p*-values of the preplanned tests of the elementary hypotheses *H_j_*, *j* ϵ *I* such that for each non-empty subset *J* ⊆ *I*, the decision function of the corresponding weighted Bonferroni test for *H_J_* is given by

(1) $$\varphi_{J}\left(p,\alpha \right)=\max_{j\in J}1_{\left\{p_{j}⩽w_{j,J}\alpha \right\}}.$$

Now, assume that midway throughout the trial, an interim analysis is performed. During the interim analysis, the data may be unblinded and trial adaptations based on internal or external information performed. To control the FWER under adaptations, an adapted closed test is defined that preserves the overall FWER. To this end we define adaptive tests for each intersection hypothesis *H_J_*, *J* ⊆ *I*. Let *J* ⊆ *I* be fixed and define for all *j* ϵ *J*

(2) Aj,J(wj,Jα)=EHJ[∣1{pj⩽wj,Jα}∣X],

where X denotes the first stage data comprised of the observations from subjects recruited in the first stage of the trial. Equation (2) is the conditional probability that the *p*-value of the preplanned test of the elementary hypothesis *H_j_* falls below its level *w*_*j,J*_ *α*, given the observed first stage data X. We refer to *A*_*j,J*_ (*w*_*j,J*_*α*) as the *partial conditional error rate* of the elementary hypothesis *H_j_* as part of intersection hypothesis *H_J_*.

Let

(3) $$B_{J}\left(\alpha \right)=\sum_{j\in J}A_{j,J}\left(w_{j,J}\alpha \right),$$

denote the sum of partial conditional error rates of those elementary hypotheses *H_j_*, *j* ϵ *J* whose intersection yields intersection hypothesis *H_J_*. As shown in Appendix B (available online as Supporting Information), any test of intersection null hypothesis *H_J_*, which may be chosen based on unblinded interim data X or external information, with a decision function ${\tilde{\varphi }}_{J}$ that satisfies

(4) $$E_{H_{J}}\left({\tilde{\varphi }}_{J}|X\right)⩽B_{J}\left(\alpha \right),$$

controls the unconditional type I error rate at level *α*, that is, $E_{H_{J}}\left({\tilde{\varphi }}_{J}\right)⩽\alpha$, assuming that the conditional expectation is uniquely defined for all X and ${\tilde{\varphi }}_{J}$. Condition (4) requires that the conditional level of the adapted test, conditional on the information used in the interim analysis assuming *H_J_*, does not exceed *B_J_*. Note that if no mid-trial adaptations are performed, condition (4) will be satisfied by the preplanned test. Therefore, in this case, the originally planned test may be performed. Furthermore, any test of hypothesis *H_J_* at level min.(*B_J_*, 1) whose test statistic is based on independent second stage observations (independent of the data of patients recruited in the first stage and independent of the choice of second stage test statistics) satisfies condition (4).

If *J* includes more than one element, in general, *B_J_* is not a probability and can take values larger than one. If *B_J_* ≥ 1, the corresponding intersection hypothesis *H_J_* can already be rejected based on the interim data, that is, ${\tilde{\varphi }}_{J}=1$. This results in an improvement of the preplanned closed test in terms of power [41].

Finally, having defined decision functions ${\tilde{\varphi }}_{J}$ of adaptive tests for all intersection null hypotheses *H_J_*, *J* ⊆ *I*, let

(5) $${\tilde{\psi }}_{i}=\min_{J\subseteq I,i\in J}{\tilde{\varphi }}_{J}$$

denote the decision function of the adaptive multiple test of the elementary hypothesis *H_i_*, *i* ϵ *I*. By the closure principle, this test controls the FWER in the strong sense. In the remainder of this manuscript we will refer to this test as adaptive graph-based multiple testing procedure (agMTP).

### 3.2. Weighted Bonferroni tests as second stage tests

One possibility to define second stage tests is to use second stage weighted Bonferroni tests that satisfy (4). Assume that at the interim analysis, some hypotheses may be dropped, the sample sizes for each of the elementary hypothesis tests may be adapted or the preplanned testing strategy modified. In principle, every second stage test satisfying (4) provides the desired FWER control. The choice of the second stage tests will, in general, depend on the adaptations performed. For example, if a dose is dropped at an interim analysis, no second stage data for the test of some of the hypotheses *H_i_*, *i* ϵ *I* will be available and this has to be accounted for when choosing intersection hypothesis tests involving such elementary hypotheses.

To construct the second stage tests define, at the interim analysis, for all elementary hypotheses *H*_*i*_, *i* ϵ *I* second stage hypothesis tests with corresponding second stage *p*-values ***q*** = (*q*_1_, …, *q*_m_). Because these tests are defined at the interim analysis, they may be based, for example, on adapted sample sizes. For notational simplicity, we also define second stage *p*-values for hypotheses where no second stage data are available, setting *q_i_* ≡ 1 in this case. We assume that under the null hypothesis, the distribution of the *q*_*i*_, *i* ϵ *I* conditional on the first stage data X is larger than or equal to the uniform distribution [0, 1] [48,49].

Define ***v***_*J*_ = (*v*_1, *J*_, …, *v*_*m*,*J*_) for all *J* ⊆ *I* with *v*_*i,J*_ = 0 for all *i* ∉ *J* and Σ_*j* ϵ *J*_ *v_j,J_* ⩽ 1. Then an adapted test of intersection hypothesis *H*_*J*_ with decision function:

(6) φ~J(q,BJ)={maxj∈J1{qj<vj,JBJ}ifBJ<11otherwise,}

satisfies (4) and, therefore, provides a level *α* test of *H_J_* regardless of mid-trial adaptations. Consequently, the corresponding closed test procedure that rejects elementary hypothesis *H_i_*, *i* ϵ *I* according to decision function

(7) $${\tilde{\psi }}_{i}=\min_{J\subseteq I,i\in J}{\tilde{\varphi }}_{J}\left(q,B_{J}\right)$$

strongly controls the FWER at level *α*. Note that for *B_J_* < 1 in Equation (6), *H_J_* is rejected if any *p*-value *q_j_*, *j* ϵ *J* is equal to or smaller than a fraction *v*_*j,J*_ of the sum of partial conditional error rates *B_J_*. Therefore, it may be interpreted as a weighted Bonferroni procedure with weights ***v**_J_* and level *B_J_*, the latter of which depends on the observed first stage data. To control the FWER, the ***v**_J_* may be chosen arbitrarily for each non-empty *J* ⊆ *I* but the choice of weights will have an impact on the power of the procedure. For example, hypotheses for which no second stage data are available such that *q_i_* ≡ 1 will be assigned weight zero in an efficient test.

#### 3.2.1. Proposals for graph-based choices of second stage weighted Bonferroni tests

An efficient and transparent way to choose the *v*_*i, J*_, *i* ϵ *I*, for all *J* ⊆ *I* can be based again on graphs. Let ${\tilde{w}}_{I},{\tilde{G}}_{I}$ denote an adapted second stage graph that is chosen based on the unblinded first stage data. This graph defines second stage weights ${\tilde{w}}_{J}=\left({\tilde{w}}_{1,J},...,{\tilde{w}}_{m,J}\right)$ for all intersection hypotheses *J* ⊂ *I* according to the algorithm in Appendix A. Especially, hypotheses *H*_*i*_ that are dropped in the interim analysis, as, for example, hypotheses corresponding to dropped treatments or sub-populations, are assigned node weight and edge weight equal to zero. Thus, no weight is assigned to these hypotheses in the second stage tests (i.e., ${\tilde{w}}_{i,J}=0$ for all *J* ⊆ *I*).

A simple (and valid, in terms of FWER control) choice of the weights *v*_*j, J*_ in (6) is to set directly $v_{j,J}={\tilde{w}}_{j,J}$ for all intersection hypotheses. However, even if we chose the original weights, that is, setting *v*_*j, J*_ = *w*_*j, J*_, the partial conditional error rates *v*_*j, J*_ *B*_*J*_ applied to the second stage elementary *p*-values in general will not correspond to the original test (i.e., *v*_*j,J*_*B*_*J*_ ≠ *A*_*j,J*_(*w*_*j,J*_*α*)). Therefore, we propose to use the weights

(8) vj,J={Aj,J(w~j,JγJ)∕BJ,ifw~j,J,BJ>0,0otherwise},

where *γ*_*J*_ is a constant that solves

(9) $$\sum_{j\in J}A_{j,J}\left({\tilde{w}}_{j,J}\gamma_{J}\right)=B_{J}.$$

Conditional on the first stage data and given the modifications to the weighting strategy, *γ*_*J*_ provides an adjusted significance level that ensures for the adapted test of *H*_*J*_ to satisfy (4). Consequently, the corresponding closed test procedure provides strong FWER control. If the weights are not modified at interim (i.e., ${\tilde{w}}_{J}=w_{J}$), the solution to Equation (9) is *γ*_*J*_ = *α* such that the resulting adapted intersection hypothesis tests use the same conditional levels for each elementary hypothesis as the preplanned test (i.e., *v*_*j, J*_ *B*_*J*_ = *A*_*j, J*_(*w*_*j, J*_ *α*)). A second stage weight ${\tilde{w}}_{j,J}=0$ results in *v*_*j, J*_ = 0 permitting, for example, to set the conditional levels applied to dropped hypotheses to zero. If the test statistics have a discrete distribution such that *A*_*j, J*_ is not continuous, (9) may not have a solution. In this case, we choose *γ*_*J*_ satisfying $\sum_{j\in J}A_{j,J}\left({\tilde{w}}_{j,J}\gamma_{J}\right)⩽B_{J}$. To distinguish between the weights ${\tilde{w}}_{j,J}$ and *v*_*j,J*_, we will refer to the latter as *conditional error allocation fractions* in the following.

##### Example 1

**Figure 2:**
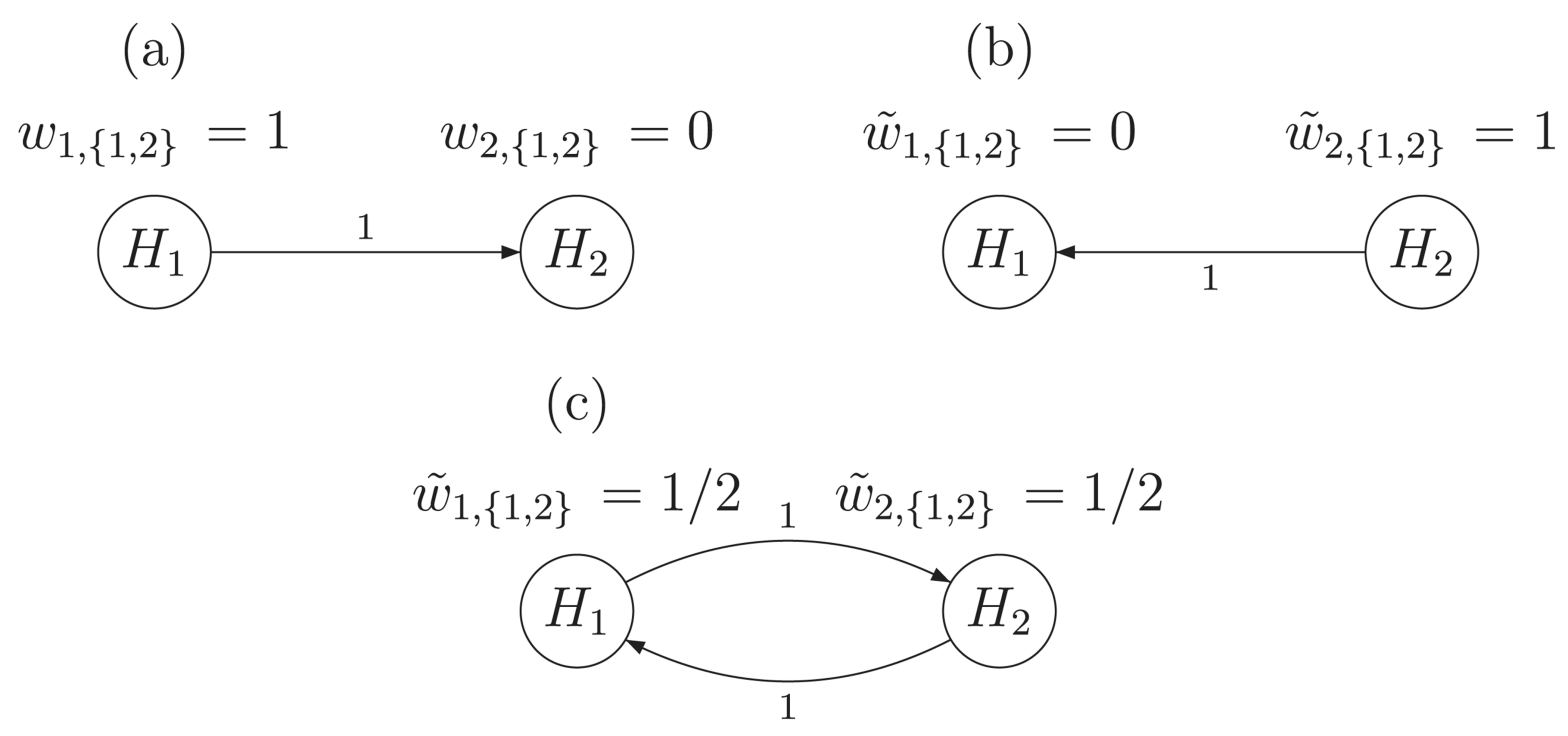
Graphical weighting procedures for (a) a hierarchical test of a primary hypothesis *H*_1_ and a secondary hypothesis *H*_2_; (b) adapted weighting procedure reversing the order of *H*_1_ and *H*_2_; and (c) adapted weighting strategy corresponding to the Bonferroni–Holm procedure.

Consider the hierarchical test of two hypotheses *H*_1_ and *H*_2_. The corresponding graph is depicted in Figure 2a. For illustrative purposes, assume that in the interim analysis, all hypotheses are continued to the second stage but it is decided to reverse the order of hypotheses in the testing strategy, resulting in the second stage graph as shown in Figure 2b. Then,

$$\begin{array}{cc} w_{I} & =\left(1,0\right),\;\;\;\;{\tilde{w}}_{I}=\left(0,1\right),\\ G_{I} & =\left(\begin{array}{cc} 0 & 1\\ 0 & 0 \end{array}\right),\;\;\;{\tilde{G}}_{I}=\left(\begin{array}{cc} 0 & 0\\ 1 & 0 \end{array}\right). \end{array}$$

There are three (intersection) null hypotheses *H*_{1,2}_, *H*_{1}_, and *H*_{2}_. The original graph results in weights ***w***_{1,2}_ = (1, 0), ***w***_{1}_ = (1, 0), and ***w***_{2}_ = (0, 1) and the modified graph in adapted weights ${\tilde{w}}_{\left\{1,2\right\}}=\left(0,1\right)$, ${\tilde{w}}_{\left\{1\right\}}=\left(1,0\right)$, and ${\tilde{w}}_{\left\{2\right\}}=\left(0,1\right)$. To compute the allocation fractions *v*_*j, J*_, note that in the intersection hypothesis test, all weights are allocated to the hypothesis with higher priority such that for the global null hypothesis *H*_{1,2}_, Equation (9) reduces to

$$A_{1,\{1,2\}}\left(0\right)+A_{2,\{1,2\}}\left(\gamma_{\left\{1,2\right\}}\right)=A_{1,\{1,2\}}\left(\alpha \right)+A_{2,\{1,2\}}\left(0\right)=B_{\left\{1,2\right\}}\left(\alpha \right),$$

and for *H*_{1}_ and *H*_{2}_, we trivially get *A*_1,{1}_ (*γ*_{1}_) = *A*_1,{1}_(*α*) and *A*_2,{2}_ (*γ*_{2}_) = *A*_2,{2}_ (*α*). As *A*_*i,J*_(0) = 0, we get *v*_1, {1,2}_ = 0, *v*_2, {1,2}_ = 1 and *v*_1, {1}_ = *v*_2, {2}_ = 1. The resulting adapted closed test rejects *H*_2_ if *q*_2_ falls below min {*A*_1,{1,2}_(*α*), *A*_2,{2}_ (*α*)}. If *H*_2_ is rejected, *H*_1_ may be rejected if *q*_1_ ⩽ *A*_1 {1}_(*α*). Depending for which hypothesis the partial conditional error rate based on the first stage observations is higher, one gets either *γ*_{1,2}_ ⩽ *α* if *A*_1,{1,2}_(*α*) ⩽ *A*_2,{1,2}_(*α*) or *γ*_{1,2}_ ≥ *α* if *A*_1,{1,2}_(*α*) ≥ *A*_2,{1,2}_ (*α*) (given that the partial conditional error rate is non-decreasing in the *α* level).

As another option for an interim design change consider that instead of reversing the order of the fixed sequence test, the weighting strategy is changed to a Bonferroni–Holm procedure. The corresponding graph is depicted in Figure 2c; edge and node weights are given by

$$\begin{array}{cc} {\tilde{w}}_{I} & =\left(1∕2,1∕2\right),\\ {\tilde{G}}_{I} & =\left(\begin{array}{cc} 0 & 1\\ 1 & 0 \end{array}\right). \end{array}$$

To compute the corresponding partial conditional error allocation fractions, the following equation has to be solved in *γ*_{1,2}_

$$A_{1,\{1,2\}}\left(\frac{\gamma_{\left\{1,2\right\}}}{2}\right)+A_{2,\{1,2\}}\left(\frac{\gamma_{\left\{1,2\right\}}}{2}\right)=A_{1,\{1,2\}}\left(\alpha \right)=B_{\left\{1,2\right\}}.$$

Consequently, the sum of conditional errors *B_J_* is split between *H*_1_ and *H*_2_ according to $v_{1,\{1,2\}}=A_{1,\{1,2\}}\left(\frac{\gamma_{\left\{1,2\right\}}}{2}\right)∕A_{1,\{1,2\}}\left(\alpha \right)$ and $v_{1,\{1,2\}}=A_{2,\{1,2\}}\left(\frac{\gamma_{\left\{1,2\right\}}}{2}\right)∕A_{1,\{1,2\}}\left(\alpha \right)$. In this case, the conditional error allocation fractions differ from the choice of second stage weights ${\tilde{w}}_{1,\{1,2\}}={\tilde{w}}_{2,\{1,2\}}=1∕2$. The specific proportions depend on the observed first stage data and the type of conditional error function. The resulting second stage test of *H*_1_ then requires that *q*_1_ ⩽ min {*A*_1,{1,2}_(*γ*_{1,2}_/2), *A*_1,{1}_(*α*)}, that of *H*_2_ that *q*_2_ ⩽ min {*A*_2,{1,2}_ (*γ*_{1,2}_/2), *A*_2,{2}_ (*α*)}. This new design permits rejection of either *H*_1_ or *H*_2_ without rejecting the other.

### 3.3. A simple, strictly conservative alternative adaptive procedure

For adaptive designs where hypotheses may be dropped in an interim analysis (for example, if treatment arms are selected) but no sample size reassessment is allowed, one can apply a simple adaptive multiple comparison procedure (saMTP) that controls the FWER in the strong sense but is strictly conservative. At the final analysis, set the *p*-values of dropped hypotheses (that cannot be tested because of lacking second stage data) to one and perform the original preplanned graph-based sequentially rejective procedure [8]. To also permit sample size reassessment, one can apply the preplanned test procedure to marginal *p*-values, of adaptive combination tests [49], again setting the *p*-values of dropped hypotheses equal to 1. For example, when testing one-sided hypothesis, the inverse normal method, $p_{j}=1-\Phi \left(\sqrt{n^{\left(1\right)}∕n}c_{1-{\tilde{q}}_{j}}+\sqrt{n-n^{\left(1\right)}∕n}c_{1-q_{j}}\right)$, gives such a *p*-value where *n*^(1)^ and *n* denote the preplanned first stage and overall groupwise sample sizes, respectively. Furthermore, ${\tilde{q}}_{j}$ and *q_j_* denote stagewise elementary *p*-values of the first and second stage tests of *H_j_* computed from the first (second, respectively) stage observations only. *Φ* and *c*_*γ*_ denote the cumulative distribution function and quantile of the standard normal distribution. The resulting adaptive procedure is equivalent to the graph-based partitioning algorithm (gPA) proposed in [40]. Note that if one-sided z-tests for the comparison of normally distributed means are preplanned and only dropping of hypotheses but no sample size reassessment is permitted, saMTP and gPA are the same procedures.

Our proposal - agMTP as defined in Section 3.1- improves gPA and saMTP in several ways: it is more flexible because it allows for interim modifications of the weighting strategy, it permits to reject intersection hypotheses at the interim analysis (whenever *B_J_* ≥ 1), and it is uniformly more powerful than the test based on the inverse normal method because it “re-uses” the partial conditional error rates of dropped hypotheses.

To show the latter, let *I*′ ⊆ *I* denote the index set of hypotheses carried forward to the final analysis and assume ∣*I*′∣, ∣*I* \ *I*′∣ > 0. First, note that gPA retains an intersection hypothesis *H*_*J*_, *J* ⊆ *I* if *J* ∩ *I*′ = θ. Otherwise, it rejects *H_J_* if for some *j* ϵ *J* ∩ *I*′, *p_j_* ⩽ *w*_*j,J*_*α*. Written as a condition on *q_j_* it is easy to see that *p_j_* ⩽ *w*_*j,J*_*α* iff

(10) $$q_{j}⩽1-\Phi \left(\sqrt{\frac{n}{n-n^{\left(1\right)}}}c_{1-w_{j,J}\alpha }-\sqrt{\frac{n^{\left(1\right)}}{n-n^{\left(1\right)}}}c_{1-{\tilde{q}}_{j}}\right).$$

In contrast, consider agMTP and consider a graph-based test using inverse normal combination tests with p-values *p_j_* as above. Then the partial conditional error rate *A*_*j,J*_(*w*_*j,J*_*α*) is equal to the right hand side of (10). Consequently, gPA rejects *H_J_* if at least one *q_j_* ⩽ *A*_*j,J*_(*w*_*j,J*_*α*) and agMTP if either *B_J_* = Σ_*j*ϵ*J*_ *A*_*j,J*_(*w*_*j,J*_*α*) ≥ 1 or (using (6)) at least one *q_j_* ⩽ *v*_*j,J*_*B_J_*. It therefore remains to show that *A*_*j, J*_ ⩽ *v*_*j,J*_*B*_*J*_ for all *j* ϵ *J* ∩ *I*′ and that the inequality is strict for some cases.

For example, one may choose partial conditional error allocation fractions

$$v_{j,J}=\left(A_{j,J}\left(w_{j,J}\alpha \right)+\frac{\sum_{i\in J\backslash I^{\prime }}A_{i,J}\left(w_{i,J}\alpha \right)}{|J\cap I^{\prime }|}\right)∕B_{J}$$

for *j* ϵ *J* ∩ *I*′ and *v*_*j, J*_ = 0 otherwise. Then $v_{j,J}B_{J}=A_{j,J}\left(w_{i,J}\alpha \right)+\frac{\sum_{i\in J\backslash I^{\prime }}A_{i,J}\left(w_{i,J}\alpha \right)}{|J\cap I^{\prime }|}\geq A_{j,J}\left(w_{j,J}\alpha \right)$ which is strictly larger if a hypothesis with positive first stage weight is dropped in the interim analysis.

Furthermore, the result also holds if the conditional error allocation fractions *v*_*j,J*_ are chosen as suggested in (8). Consider that the second stage weights ${\tilde{w}}_{j,J}$ are set identical to the first stage weights *w*_*j,J*_ for *j* ϵ *I*′ and set to zero (i.e., ${\tilde{w}}_{j,J}=0$) otherwise. Then, the conditional error allocation fractions *v*_*j,J*_ proposed in Equation (8) are zero for *j* ϵ *J* \ *I*′ and otherwise satisfy

$$\begin{array}{cc} \sum_{j\in J\cap I^{\prime }}v_{j,J}B_{J} & =\sum_{j\in J\cap I^{\prime }}A_{j,J}\left(w_{j,J}\gamma_{J}\right)\\ & =\sum_{j\in J\cap I^{\prime }}A_{j,J}\left(w_{j,J}\alpha \right)+\sum_{j\in J\backslash I^{\prime }}A_{j,J}\left(w_{j,J}\alpha \right) \end{array}$$

which implies *γ*_*J*_ ≥ *α* and consequently *v*_*j,J*_*B*_*J*_ = *A*_*j, J*_(*w*_*j,J*_*γ*_*J*_) ≥ *A*_*j, J*_(*w*_*j,J*_*α*) for all *j* ϵ *J* ∩ *I*′ and the inequality is strict if any *w*_*j,J*_ > 0 for some *j* ϵ *J* \ *I*′.

In contrast to gPA and saMTP, agMTP is in general not consonant, even if a consonant multiple test procedure is preplanned. For example, consider a test of two hypotheses *H*_1_ and *H*_2_ and that *H*_2_ is dropped at interim and the second stage tests are defined as in Section 3.2. The conditional level of the test of intersection hypothesis *H*_1_ ∩ *H*_2_ is *B*_{1,2}_ = *A*_1,{1,2}_(w_1,{1,2}_*α*) + *A*_2,{1,2}_ (*w*_2,{1,2}_*α*), which may be larger than *A*_1,{1}_(*α*) (the conditional level for the test of *H*_1_). Consequently, by setting ${\tilde{w}}_{\left\{1,2\right\}}=\left(1,0\right)$ we have $A_{1,\{1,2\}}\left({\tilde{w}}_{1,\{1,2\}}\right)>A_{1,\{1\}}\left(\alpha \right)$, such that *H*_1_ ∩ *H*_2_ may be rejected but no elementary hypothesis. Even if no interim adaptations are performed, a non-consonant test procedure may result. For example, if *B*_{1,2}_ ≥ 1 one may reject *H*_{1,2}_ at interim, however both second stage p-values *q_i_* may be larger than the corresponding partial conditional error rates *A*_*i*,{*i*}_ (*α*), such that no elementary hypothesis may be rejected. As a consequence all 2*^m^* – 1 intersection hypothesis tests have to be performed, which for large numbers of hypotheses becomes computationally infeasible. Since saMTP and gPA are consonant a sequentially rejective algorithm requiring at most *m* steps can be applied. Thus, there is a trade-off between the power advantage and computational costs.

## 4. CASE STUDY

### 4.1. Preplanned design

To demonstrate the practical application of the presented methodology, consider a clinical trial in the spirit of the multiple sclerosis study investigated in [8]. In this case study, two treatment regimens with a new therapeutic agent (Treatment 1: 300*μg* three times a day, Treatment 2: 900 *μg* once daily) are compared to a control treatment in a parallel group design. For each test treatment two hierarchically ordered endpoints (annualized relapse rate followed by number of lesions in the brain) are compared to control. In total four one-sided elementary null hypotheses *H_i_* : *θ_i_* ⩽ 0 are tested, where *θ*_1_, *θ*_2_ refer to the treatment effect differences (compared to control) of treatments 1 and 2 in the primary endpoint and *θ*_3_, *θ*_4_ to the treatment effect differences in the secondary endpoint, respectively. The FWER is to be controlled at the one-sided level *α* = 0.025. The planned per-group sample size *n* is assumed to be large enough such that the *z*-test for the comparison of normally distributed means gives conservative elementary p-values *p_i_*. Based on the clinical relevance of the endpoints and the nature of the test treatments, a multiple comparison procedure with the following properties is proposed:

(1) The testing strategy should be symmetric in the two treatment regimens because based on prior knowledge each is equally likely to be effective. Assuming equal effect sizes, the statistical power should be the same for both treatment control comparisons.
(2) Testing the primary endpoint takes precedence over testing the secondary endpoint. Unless superiority of a treatment with regard to the primary endpoint can be shown, inference on the treatments efficacy regarding the secondary end point is not of interest.

**Figure 3:**
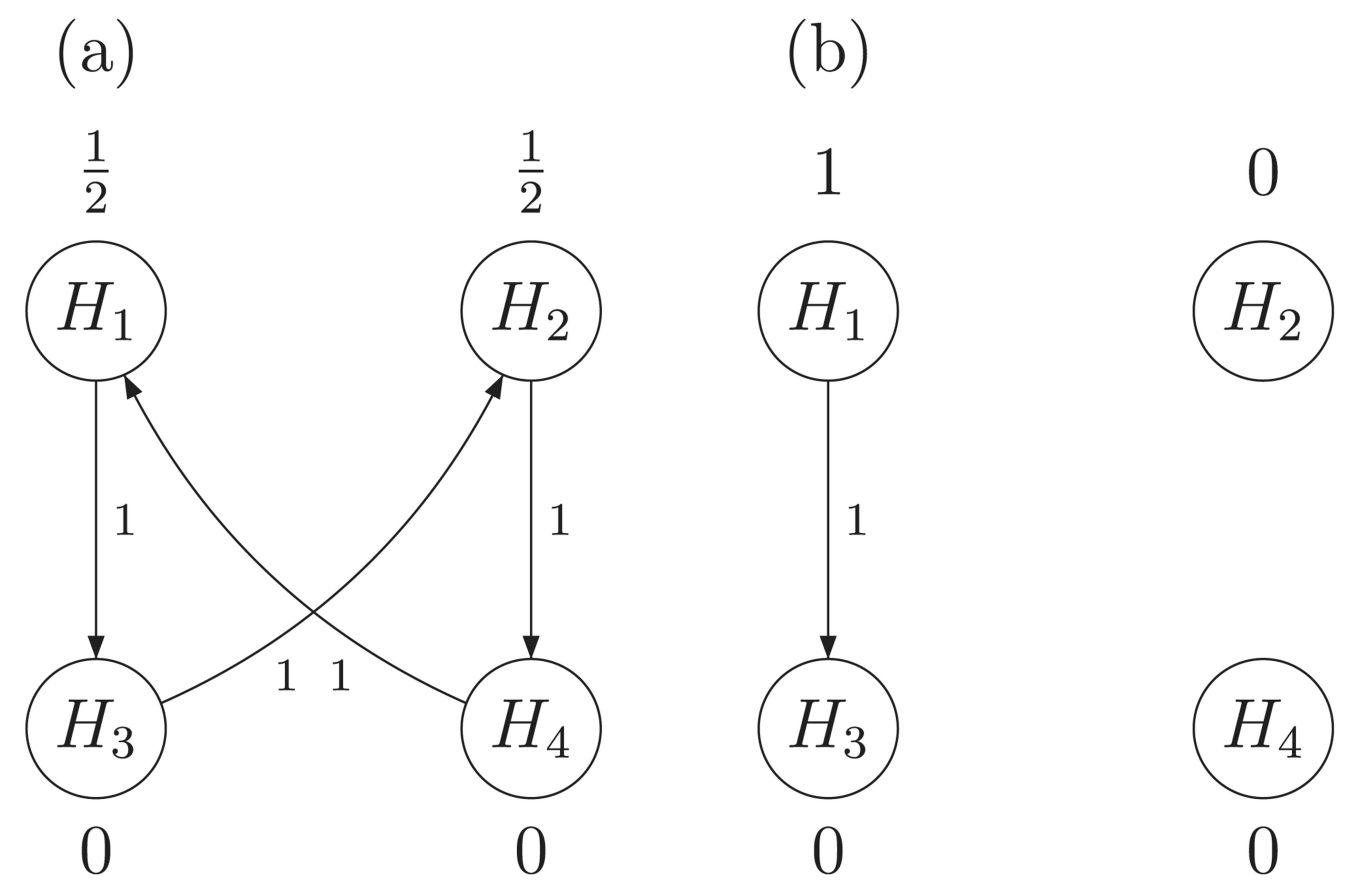
(a) Graph defining the multiple testing procedure of the multiple sclerosis trial in Sections 4 and 5. (b) Modified graphical weighting strategy - if Treatment 2 (*i.e., H*_2_, *H*_4_) is dropped at interim.

A multiple comparison procedure with the desired properties is specified by the graph in Figure 3a. The four hypotheses are represented by nodes in the graph. Each node is allocated an initial weight giving the portion of the overall *α* level that is used in the test of the intersection of all elementary hypotheses represented in the graph. To reflect the prioritization of the primary endpoint, initially the full *α*-level is distributed between the hypotheses of efficacy in the primary endpoint. Table I lists the weights *w*_*j,J*_ of all intersection hypotheses tests as defined by the graph. The closure of the corresponding weighted Bonferroni intersection hypothesis tests is equivalent to a sequentially rejective test where initially only *H*_1_ and *H*_2_ are tested at levels *α*/2, whereas *H*_3_, *H*_4_ are allocated weight zero. If one of the primary hypotheses can be rejected, its level is reallocated to the corresponding secondary hypothesis. If, for a treatment arm both hypotheses can be rejected, the primary hypothesis (and given it can be rejected also the secondary hypothesis) can be tested at full level *α*.

### 4.2. Design modification after an adaptive interim analysis

Assume that after *n*^(1)^ = *n*/2 patients in each group have been recruited, an unblinded interim analysis is performed. Let $z_{1}^{\left(1\right)}=1.66,z_{2}^{\left(1\right)}=1.42,z_{3}^{\left(1\right)}=1.90,z_{4}^{\left(1\right)}=.79$ denote the first stage standardized mean differences of the treatment-control comparisons corresponding to the hypotheses *H*_1_, …, *H*_4_. After inspection of the unblinded safety data, concerns regarding the safety of treatment regimen 2 are raised. Since, in addition, a larger interim effect size is observed for treatment regimen 1, the data safety committee decides to discontinue treatment arm 2 and to reallocated the remaining patients that were intended to be recruited for treatment arm 2 to the two remaining arms. Besides the dropping of the treatment arm and sample size reallocation, a second stage testing strategy also needs to be specified. As the treatment arm 2 has been dropped, in the final analysis only the two hypotheses regarding treatment arm 1 shall be tested. The corresponding second stage weighting strategy is defined according to the graph depicted in Figure 3b. The second stage weights ${\tilde{w}}_{j,J}=0,j\in 2,4$ for the weights corresponding to the dropped hypotheses *H*_2_ and *H*_4_ are set to zero for all *J* ⊆ {1, 2, 3, 4}. Table I lists the corresponding second stage weights ${\tilde{w}}_{j,J}$ for all intersection hypotheses. Finally, assume that it is planned to again apply marginal *z*-tests to the second stage data.

### 4.3. Final analysis

Assume that the observations collected from subjects recruited in the second stage yield second stage *z*-scores $z_{1}^{\left(2\right)}=1.56$ and $z_{3}^{\left(2\right)}=1.87$, which are computed fromthe observations collected in the second stage only, corresponding to second stage p-values, *q*_1_ = 0.059 and *q*_3_ = 0.031. To construct the adaptive test for the final analysis, for all 15 intersection hypotheses *H*_*J*_ *J* ⊆ {1, 2, 3, 4} the sums of the partial conditional error rates are computed. Let *J* ⊂ *I* and *j* ϵ *J*. The partial conditional error rate of the z-test is given by

(11) $$\begin{array}{cc} A_{j,J}\left(w_{j,J}\alpha \right) & =P\left(Z_{j}>c_{1-w_{j,J}}\alpha |z_{j}^{\left(1\right)}\right)\\ & =1-\Phi \left(\frac{c_{1-w_{j,J}}\alpha -z_{j}^{\left(1\right)}\sqrt{\frac{n^{\left(1\right)}}{n}}}{\sqrt{1-\frac{n^{\left(1\right)}}{n}}}\right), \end{array}$$

where *Z*_*j*_ denotes the z-statistics of the fixed sample z-test for *H*_*j*_ with a preplanned sample size of *n* observations per group. For example for the global null hypothesis *H*{1,2,3,4} plugging $z_{1}^{\left(1\right)}$ and $z_{2}^{\left(1\right)}$ into (11) we get

$$A_{1,\{1,2,3,4\}}\left(\alpha ∕2\right)=1-\Phi \left(\frac{2.24-1.66\sqrt{.5}}{\sqrt{.5}}\right)=0.066,$$

and

$$A_{2,\{1,2,3,4\}}\left(\alpha ∕2\right)=1-\Phi \left(\frac{2.24-1.42\sqrt{.5}}{\sqrt{.5}}\right)=0.04.$$

Since *w*_3,{1,2,3,4}_ = *w*_4,{1,2,3,4}_ = 0, the corresponding partial conditional errors are zero, as well. Table I lists these partial conditional errors and their sums *B*_*J*_ for the second stage tests for each intersection hypothesis. Because for each intersection hypothesis only one of the weights is positive, the conditional error allocation fractions *v_j,J_* defined in (8) coincide with the second stage weights ${\tilde{w}}_{j,J}$ in this example. Therefore, the resulting adaptive test rejects *H*_1_, if *q*_1_ falls below the minimum of the sums of partial conditional error rates *B*_*J*_ for all *J* ⊆ {1, 2, 3, 4} with 1 ϵ *J*. Because of the hierarchical structure of the underlying graph, to reject *H*_3_, *additionally q*_3_ needs to fall below the minimum of *B*_J_ for all *J* ⊆ {1, 2, 3, 4} with 3 ϵ *J* and 1 ∉ *J*. Consequently, according to Table I the critical level for *q*_1_ is 0.075 and to additionally reject *H*_3_, *q*_3_ needs to fall below 0.088. Hence, in this example both hypotheses are rejected. The adaptive procedure for the *z*-test has been implemented by the first author as part of the R-package gMCP Version 0.8-7 [50]. For the R-code to replicate the calculations of the case study see Appendix C (available online as Supporting Information).

## 5. SIMULATION STUDY

Based on a simulation study we investigated the operating characteristics of the agMTP with second stage weights as proposed in Section 3.2 for a range of distributional assumptions and compare them with the gPA by [40], which is described in Section 3.3. The setting of the simulation study is similar to that of the case study in Section 4: a three armed clinical trial comparing two treatments with a common control using a primary and a secondary endpoint. Additionally we simulated a toxicity marker, which is positively correlated with efficacy in the primary endpoint.

In the simulations an interim analysis is performed after half of the observations have been collected and one of four interim adaptation rules is applied.

**Preplanned (PP):** perform no adaptations; complete the trial as planned and at the final analysis test all four elementary null hypotheses as initially planned.

**Select better (SB):** select the treatment arm with higher observed interim efficacy estimate and at the final analysis test only the hypotheses corresponding to the selected treatment.

**50:50 (FF):** randomly (with equal probability and independent of the outcomes) select either treatment arm 1 or 2, drop the other and at the final analysis test only hypotheses corresponding to the selected treatment.

**Safety (SF):** if the estimate of the toxicity marker for a treatment exceeds a certain level *s*, drop the corresponding treatment arm, otherwise, perform no adaptations.

Rule *Preplanned* represents the baseline scenario of a fixed sample trial without any adaptation. Rule *Select better* represents a simple adaptation rule, where the interim decision relies on efficacy data only. Rule *50:50* reflects the complexity of the decision process when it comes to choosing a treatment in reality, where the decision may also depend on other, possibly external, factors than those provided by a few well defined endpoints. Rule *Safety* represents a scenario where the interim decision is driven by safety considerations. For all adaptation rules that drop treatments at interim, trials were simulated with and without sample size reallocation, where in the latter scenario patients preplanned for the dropped treatment arm are equally allocated to the remaining treatment arm and the control group. For agMTP, as in the case study, the second stage weights corresponding to the dropped treatment are set to zero; of the continued treatment to one. Note that in the case that no sample size reallocation is performed, the gPA is equivalent to the simple adaptive multiple testing procedure discussed in the case study in Section 4.

We assume that observations follow a multivariate normal distribution with known variances. Then, in the preplanned trial, with *n* patients per-treatment arm, the standardized treatment–control differences of the primary and secondary endpoints, *z_i_*, *i* ϵ 1, …, 4, and of the toxicity markers, *t*_1_, *t*_2_, are multivariate normal with mean vector

$$\theta =\sqrt{n∕2}\left(\delta_{1}∕\sigma_{1},\delta_{2}∕\sigma_{2},\delta_{1}∕\sigma_{1},\delta_{2}∕\sigma_{2},0,\kappa ∕\sigma_{t}\right),$$

where *δ*_1_, *δ*_2_ (*σ*_1_, *σ*_2_) denote the mean effect sizes (and standard deviations) for the efficacy for Treatments 1 and 2, respectively. The effect sizes of the toxicity markers are 0 for Treatment 1 and *κ* for Treatment 2 (with common standard deviation 1). The standardized effect sizes for the primary and secondary endpoints are assumed to be equal within each treatment group. Because sample sizes are assumed to be balanced, the correlation between test statistics for the same endpoint is 1/2. We denote the correlation between endpoints within a treatment arm by *ρ* and assume them to be equal for either treatment. We assume that the toxicity markers have equal correlation *ζ* with the corresponding primary endpoint. The correlation matrix of (*z*_1_, *z*_2_, *z*_3_, *z*_4_, *t*_1_, *t*_2_) is then given by

$$\Sigma =\left(\begin{array}{cccccc} 1 & 1∕2 & \rho & \rho ∕2 & \zeta & \zeta ∕2\\ 1∕2 & 1 & \rho ∕2 & \rho & \zeta ∕2 & \zeta \\ \rho & \rho ∕2 & 1 & 1∕2 & \zeta \rho & \zeta \rho ∕2\\ \rho ∕2 & \rho & 1∕2 & 1 & \zeta \rho ∕2 & \zeta \rho \\ \zeta & \zeta ∕2 & \zeta \rho & \zeta \rho 2 & 1 & 1∕2\\ \zeta ∕2 & \zeta & \zeta \rho ∕2 & \zeta \rho & 1∕2 & 1 \end{array}\right).$$

Note that knowledge of *ρ* and *ζ* is not required to implement the multiple test procedure, but they need to be specified for the simulation study. We assume that an interim analysis is performed after *n*^(1)^ = *n*/2 patients per group have been observed. Consequently, the first stage test statistics follow a multivariate normal distribution as specified earlier, replacing *n* by *n*^(1)^.

For the simulation study, we considered a common standard deviation of *σ*_1_ = *σ*_2_ = 1, correlation coefficients *ρ* = 0.3, and *ζ* = 0.5. We chose the preplanned per-group sample size to provide at least 90% power to reject any primary hypothesis using the fixed-sample graph-based test, as defined by Figure 3a and assuming equal effect sizes for both treatments and endpoints, that is, *δ*_1_ = *δ*_2_ = 0.4. We, further, require that the sample sizes are divisible by 4 to be able to reallocate half of the second stage sample size. Using the function extractPower from GNU R package gMCP [50], we computed the smallest preplanned sample size *n* = 116 per group that satisfies these requirements. The edited sentence is incorrect. This results in the first and second stage sample sizes of 58 per treatment group and stage, if no sample size reallocation is performed and the second stage sample size of 82 for the selected treatment, if sample size reallocation is performed.

The simulation study covers a range of distributional scenarios: no effect in any treatment arm (*δ*_1_ = *δ*_2_ = 0), equal effect sizes in both treatment arms (*δ*_1_ = *δ*_2_ = 0.4), a smaller effect size in one treatment arm (*δ*_1_ = 0.3, *δ*_2_ = 0.4), and a positive effect in two treatment arms only (*δ*_1_ = 0, *δ*_2_ = 0.4). For all safety scenarios (rule SF), the threshold for the toxicity markers (*t*_1_, *t*_2_) was set to the 95% quantile of the standard normal distribution (i.e., *s* = 1.645). For all configurations of effect sizes, we simulated safety scenarios with toxicity effects *κ* = 0.2 and *κ* = 0.4. All simulations were implemented using R [51] and 10^6^ simulation runs per scenario (simulation standard error < 0.0005). Simulation code is available at request from the authors.

The results of our simulation study are summarized in Table II. There, we present the probabilities to reject at least one null hypothesis (*π*), to reject a particular null hypothesis *H_i_* (*π_i_*), and to drop treatment arm *i* (*η_i_*). Under the global null hypotheses (i.e., *δ*_1_ = *δ*_2_ = 0), *π* denotes the FWER and for *δ*_1_ = 0, *δ*_2_ = 0.4, *π* combines erroneous rejections of *H*_1_ with correct rejections of *H*_2_. Accordingly, *π*_1_ and *π*_3_ give Type I error rates, *π*_2_ and *π*_4_ powers. For the remaining scenarios, all null hypotheses are false and the probabilities correspond to the power.

The results of the simulation study show that agMTP is more powerful than saMTP and gPA and thereby confirm the theoretical results of Section 3.3. For the scenarios shown in Table II, the overall power *π* is improved by up to 5 percentage points; the power to reject a particular hypothesis *π_i_* is improved by up to 7 percentage points. The largest improvements are achieved in scenarios where an efficacious treatment is dropped, for example, due to safety reasons. This is illustrated by the results for the selection rules FF and SF. For scenarios under the global null hypothesis, agMTP is less conservative than saMTP and gPA.

Selecting the treatment with the larger interim effect and performing a sample size, reallocation (rule SB) is a very promising adaptive strategy as far as the overall power *π* is concerned. With these adaptations, agMTP yields even larger overall power than the preplanned design (rule PP). Although both designs have the same overall sample sizes, power is improved by 4–8 percentage points. The power *π_i_* to reject a particular hypothesis *H_i_* and the number of rejected hypotheses, however, is decreased because of dropping hypotheses already at interim. If only the more promising treatment arm is continued at interim without sample size reallocation (rule SB, numbers in brackets), the loss of primary power P does not exceed 2 percentage points compared with the preplanned design (rule PP), which uses a 20% larger overall sample size. This also shows that sample size reallocation (rule SB) increases the power substantially compared with adaptive trials without sample size reallocation (SB, numbers in brackets).

For scenarios where the non-efficacious treatment is dropped (SB), the power advantage of agMTP compared with saMTP and gPA is less than 1 percentage point. But the power advantage of agMTP over gPA and saMTP is larger if the sample sizes are held fixed (SB, numbers in brackets). Considering the theoretical results in Section 4, it is not surprising that our procedures are most advantageous in scenarios where an efficacious treatment is dropped (rules FF, SF). In this case, promising interim results for the dropped treatment will lead to a corresponding large partial conditional error rate that may be reused. If only hypotheses with low partial conditional errors are dropped, little can be gained by recycling partial conditional errors in the second stage. Overall, sample size reallocation leads to large improvements of power only in scenario SF with *κ* = 0.4 (and to a lesser extent for *κ* = 0.2), where the second treatment is dropped in the majority of cases, whereas the first treatment is dropped only rarely; the advantage of sample size reallocation on *π*_2_ and *π*_4_ is hardly noticeable.

## 6. DISCUSSION

In this paper, we generalize graph-based multiple testing procedures to flexible designs that allow for an adaptation of the trial design after an unblinded interim analysis. The proposed graph-based adaptive testing procedures can be tailored to reflect the structure and logical relations between hypotheses and control the FWER in the strong sense. The approach covers a large class of procedures including (parallel) gatekeeping, fixed sequence, and fallback tests. Although the adaptive tests are based on partial conditional error rates and can be applied to all multiple testing procedures based on weighted Bonferroni tests, the use of graphs to specify the weights in the planning phase as well as in the interim analysis allows for an intuitive communication of the testing strategy. Examples of adaptations in clinical trials are the modification of the testing strategy, sample size reassessment, modification of endpoints, dropping of treatment arms, or subgroups. The latter implies that hypotheses are dropped at the interim analysis. Similar as in [30], the procedure can also be extended to allow for the addition of new hypotheses at the interim analysis.

For the implementation of the adaptive test, the joint distribution of the elementary test statistics need not be known. Only the marginal distributions of the data for each elementary test statistics need to be specified under the null hypothesis in order to compute the partial conditional error rates. Therefore, the procedure can also be applied in settings where different types of statistics are used to test the different elementary hypotheses. For example, the primary hypothesis may concern a metric endpoint, whereas the secondary endpoint is binary. In the case study, we demonstrated the computation of the partial conditional error rates of the *z*-test. In such a setting where the marginal distribution of the observations is fully specified by the null hypothesis, the conditional error can be directly calculated. For settings with nuisance parameters, the partial conditional error rates can often be approximated based on asymptotic results [41,52]. Especially, the *z*-test approximation can be applied for various statistical tests similar as in group sequential designs. An alternative to asymptotic approximations is the application of *p*-value combination tests to define the marginal tests. For example, if, instead of standard fixed sample test statistics for each elementary hypothesis, a test based on the weighted inverse normal method [53] is preplanned that combines stagewise *p*-values by a weighted sum of their standard normal quantiles; the partial conditional error rate no longer depends on the nuisance parameters [54].

The adaptive procedure can be generalized to designs with more than two stages. This allows adaptations to be performed at more than one interim analyses and can be implemented by recursive application of the adaptive test as in [46]. Especially, intersection hypothesis tests can be improved if the partial conditional error rates are computed after each observation and the intersection hypothesis is rejected if the sum of the partial conditional error rates exceeds 1. Posch *et al.* [52] showed that under suitable assumption, this test asymptotically exhausts the *α* level regardless of the joint distribution and therefore improves the strictly conservative weighted Bonferroni test. The comparison of such strategies to other alternative multiple testing procedures that accounts for correlations will be part of our future research.

The proposed approach can be extended to group sequential designs for testing multiple hypotheses, which permit early rejection of elementary hypotheses at predefined interim analyses. This can be implemented by applying the partial conditional error rate approach to the group sequential graph-based multiple testing procedures proposed in [55]. In this setting, the derivation of corresponding second stage tests will require additional considerations. For example, how to choose (group sequential) adapted tests that reflect the intention of the (potentially modified) weighting strategy and adhere to the (potentially modified) functional form of the desired critical boundaries (e.g., Pocock or O’Brien–Fleming type boundaries), how to deal with the possibility that test decisions made at earlier stages are reversed at later stages, and how to decide whether or not to to stop a trial in which some but not all hypotheses are rejected early. A comprehensive treatment of these topics goes beyond the scope of this article and is part of our future research.

In the simulation study in Section 5, we assumed that a treatment arm is dropped based on safety issues observed in the interim analysis. If the toxicity marker is independent of the efficacy endpoint and only the toxicity data are used for the treatment selection, any multiple test procedure for the two remaining hypotheses (disregarding the other two initially considered hypotheses) controls the FWER. König *et al.* [15] showed that for a hierarchical test, this results in a strictly conservative test if toxicity is positively correlated to the efficacy data (i.e., on average patients that experience a larger treatment effect in the primary endpoint also experience more toxic effects). The proposed adaptive closed test procedure provides strong FWER control without any assumptions on the correlation of toxicity and efficacy endpoints and the rule for dropping treatment arms—that is, even if toxicity is negatively correlated to efficacy and/or efficacy data are used for the treatment selection.

From a purely statistical point of view, the conditional error principle guarantees strict type 1 error control even if the adaptive interim analysis is performed at a data-dependent time point, which is not prespecified. Such a flexibility is astonishing and frightening at the same time. Because in actual clinical trials, the impact of interim analyses may go beyond what is covered by the statistical model, looking at the unblinded data too frequently is not recommended. For example, leaking interim information of the treatment effect may lead to an uncontrolled change in the assessment of endpoints, the placebo effect, or the characteristics of patients recruited after the interim analyses. Therefore, to maintain the confirmatory nature of a clinical trial, details of the planned adaptations should be laid down in the study protocol and procedures to ensure the confidentiality of the interim results needed to be put in place. Furthermore, too many adaptations are likely to compromise the persuasiveness of the results. In addition, adaptations do not necessarily lead to an increased efficiency of the test procedure but may lead to unfavorable operating characteristics for the situation at hand. For example, one may be misguided by highly variable interim data based on small samples leading to inefficient changes to the study design [23]. Therefore, careful planning and evaluation of different testing strategies and scenarios is essential.

## Supplementary Material

Appendix

## Figures and Tables

**Table I:** First stage weights *w*_*j,J*_, resulting partial conditional error rates *A*_*j,J*_ and modified second stage weights ${\tilde{w}}_{j,J}$ defined via the graphs in Figure 3a,b, respectively. The last column shows the sums of the partial conditional error rates *B_J_*.

	*w*_1,*J*_	*A*_1,*J*_	${\tilde{w}}_{1,J}$	*w*_2,*J*_	*A*_2,*J*_	${\tilde{w}}_{2,J}$	*w*_3,*J*_	*A*_3,*J*_	${\tilde{w}}_{3,J}$	*w*_4,*J*_	*A*_4,*J*_	${\tilde{w}}_{4,J}$	*B_J_*
*H*_{1,2,3,4}_	½	0.066	1	½	0.04	0	0	0	0	0	0	0	0.106
*H*_{1,2,3}_	½	0.066	1	½	0.04	0	0	0	0	—	—	—	0.106
*H*_{1,2,4}_	½	0.066	1	½	0.04	0	—	—	—	0	0	0	0.106
*H*_{1,3,4}_	½	0.066	1	—	—	—	0	0	0	½	0.009	0	0.074
*H*_{2,3,4}_	—	—	—	½	0.04	0	½	0.102	1	0	0	0	0.142
*H*_{1,2}_	½	0.066	1	½	0.04	0	—	—	—	—	—	—	0.106
*H*_{1,3}_	1	0.133	1	—	—	—	0	0	0	—	—	—	0.133
*H*_{1,4}_	½	0.066	1	—	—	—	—	—	—	½	0.009	0	0.074
*H*_{2,3}_	—	—	—	½	0.04	0	½	0.102	1	—	—	—	0.142
*H*_{2,4}_	—	—	—	1	0.088	0	—	—	—	0	0	0	0.088
*H*_{3,4}_	—	—	—	—	—	—	½	0.102	1	½	0.009	0	0.111
*H*_{1}_	1	0.133	1	—	—	—	—	—	—	—	—	—	0.133
*H*_{2}_	—	—	—	1	0.088	0	—	—	—	—	—	—	0.088
*H*_{3}_	—	—	—	—	—	—	1	0.192	1	—	—	—	0.192
*H*_{4}_	—	—	—	—	—	—	—	—	—	1	0.024	0	0.024

**Table II:** Probabilities in percent: *π* to reject at least one null hypothesis, *π*_*i*_ to reject a particular hypothesis *H*_*i*_, and *η*_*i*_ to drop treatment arm *i* at interim. Numbers in brackets give rejection probabilities if no sample size reallocation is performed. 10^6^ trials were simulated assuming mean difference *δ*_*i*_ for treatment *i* (equal across endpoints) and mean toxicity response *κ* in treatment arm 2. In each scenario, the fixed sample gMCP was applied to the preplanned design. Adaptive trials were simulated applying the rules SB, 50:50 (FF), or SF. These were simulated with and without sample size reallocation of dropped treatment arms and evaluated using the agMTP and the gPA.

(*δ*_1_, *δ*_2_)	Rule(*κ*)	Procedure	* π *	* π *_1_	* π *_2_	* π *_3_	* π *_4_	* η *_1_	* η *_2_
(0, 0)	PP	gMCP	2.3	1.3	1.3	0.1	0.1	0	0
	SB	agMTP	2.2 (2.2)	1.1 (1.1)	1.1 (1.1)	0.1 (0.1)	0.1 (0.1)	50	50
		gPA	2.1 (2.1)	1.0 (1.0)	1.0 (1.0)	0.1 (0.1)	0.1 (0.1)	50	50
	FF	agMTP	1.4 (1.4)	0.7 (0.7)	0.7 (0.7)	0.1 (0.1)	0.1 (0.1)	50	50
		gPA	1.2 (1.2)	0.6 (0.6)	0.6 (0.6)	0.0 (0.0)	0.0 (0.0)	50	50
	SF(0.2)	agMTP	1.4 (1.4)	1.0 (1.0)	0.5 (0.5)	0.1 (0.1)	0.0 (0.0)	5	29
		gPA	1.4 (1.4)	1.0 (1.0)	0.4 (0.5)	0.1 (0.1)	0.0 (0.0)	5	29
	SF(0.4)	agMTP	1.2 (1.2)	1.1 (1.1)	0.1 (0.1)	0.1 (0.1)	0.0 (0.0)	5	70
		gPA	1.1 (1.1)	1.0 (1.0)	0.1 (0.1)	0.1 (0.1)	0.0 (0.0)	5	70
(0, 0.4)	PP	gMCP	79.0	2.3	78.9	0.2	65.1	0	0
	SB	agMTP	86.7 (78.6)	0.2 (0.2)	86.6 (78.4)	0.0 (0.0)	77.5 (64.9)	98	2
		gPA	86.6 (78.3)	0.1 (0.1)	86.4 (78.2)	0.0 (0.0)	77.2 (64.5)	98	2
	FF	agMTP	45.0 (40.8)	1.2 (1.2)	43.8 (39.6)	0.1 (0.1)	39.2 (32.8)	50	50
		gPA	44.4 (40.1)	0.6 (0.6)	43.7 (39.5)	0.0 (0.0)	39.0 (32.6)	50	50
	SF(0.2)	agMTP	54.2 (54.0)	1.9 (1.8)	53.4 (53.3)	0.2 (0.2)	43.7 (43.5)	5	28
		gPA	53.8 (53.7)	1.5 (1.5)	53.4 (53.3)	0.1 (0.1)	43.7 (43.5)	5	28
	SF(0.4)	agMTP	21.7 (21.7)	1.9 (1.9)	20.2 (20.2)	0.2 (0.2)	16.3 (16.3)	5	70
		gPA	21.0 (21.0)	1.1 (1.2)	20.2 (20.2)	0.1 (0.1)	16.3 (16.3)	5	70
(0.3, 0.4)	PP	gMCP	85.1	64.4	80.7	46.8	68.0	0	0
	SB	agMTP	90.0 (83.3)	28.1 (25.5)	61.9 (57.8)	22.7 (18.8)	56.9 (49.7)	67	33
		gPA	88.4 (80.9)	27.2 (24.3)	61.2 (56.6)	20.5 (16.4)	55.0 (47.1)	67	33
	FF	agMTP	82.7 (74.2)	37.6 (32.9)	45.1 (41.4)	30.2 (23.9)	41.4 (35.5)	50	50
		gPA	77.8 (68.4)	34.1 (28.9)	43.7 (39.5)	25.2 (19.0)	39.0 (32.6)	50	50
	SF(0.2)	agMTP	78.7 (76.4)	62.4 (60.2)	54.9 (54.8)	46.8 (43.8)	45.9 (45.7)	5	28
		gPA	76.8 (74.2)	60.6 (58.1)	54.8 (54.7)	44.1 (40.9)	45.8 (45.6)	5	28
	SF(0.4)	agMTP	73.2 (67.1)	66.9 (60.8)	21.0 (21.0)	52.3 (44.3)	17.4 (17.3)	5	69
		gPA	68.4 (61.7)	62.1 (55.4)	21.0 (21.0)	45.5 (37.4)	17.4 (17.3)	5	69
(0.4, 0.4)	PP	gMCP	90.6	82.6	82.7	71.2	71.2	0	0
	SB	agMTP	94.4 (89.2)	47.2 (44.6)	47.2 (44.6)	43.8 (38.9)	43.8 (38.9)	50	50
		gPA	93.2 (87.1)	46.6 (43.5)	46.6 (43.6)	42.0 (36.4)	42.0 (36.4)	50	50
	FF	agMTP	91.0 (84.0)	45.5 (42.0)	45.5 (42.0)	42.0 (36.5)	42.0 (36.4)	50	50
		gPA	87.4 (78.9)	43.7 (39.5)	43.7 (39.4)	39.0 (32.5)	39.0 (32.5)	50	50
	SF(0.2)	agMTP	86.7 (85.2)	79.5 (78.1)	56.5 (56.4)	69.8 (67.3)	48.3 (48.2)	5	29
		gPA	85.8 (83.9)	78.7 (76.9)	56.4 (56.3)	68.3 (65.2)	48.2 (48.0)	5	29
	SF(0.4)	agMTP	85.6 (81.4)	82.9 (78.8)	21.9 (21.9)	75.0 (68.1)	18.5 (18.5)	5	69
		gPA	83.2 (78.0)	80.6 (75.4)	21.9 (21.9)	70.9 (62.8)	18.5 (18.5)	5	69

gMCP, graphical multiple comparison procedure; SB, select better; SF, safety; agMTP, adaptive graph-based multiple testing procedure; gPA, graph-based partitioning algorithm.
