# Supplementary material for: Adaptive graph-based multiple testing procedures
Source: Pharm Stat. Author manuscript; Available in PMC 2016 Mar 14. (PMC4789493; doi:10.1002/pst.1640)
Supplement: Appendix [file NIHMS67343-supplement-Label.pdf]

APPENDIX

A Graphical weighting algorithm

Consider a weighted directed graph with m nodes corresponding to the m elementary null hypotheses H_i $i \in I = \{1, ...m\}$ as defined in Section 2. Given initial weights $w_{i,I}$ and matrix of edge-weights $G_I = (g_{ij,I})_{i,j\in I}$ the update rule that calculates node weights $\boldsymbol{w}_{J\setminus\{i\}}$ from $\boldsymbol{w}_J$ for non-empty subsets $J \subseteq I, i \in J$ (that contain at least one more element in addition to i) is given by

$$w_{j,J\setminus i} = \begin{cases} w_{j,J} + w_{i,J}\, g_{ij,J} & i \neq j \in J \\ 0, & \text{otherwise} \end{cases}. \tag{12}$$

The updated edge-weights $G_J = \left(g_{ij,J\setminus\{i\}}\right)_{i,j\in J\setminus\{i\}}$ are computed by

$$g_{j\ell,J\setminus i} = \begin{cases} \frac{g_{j\ell,J}+g_{ji,J}g_{i\ell,J}}{1-g_{ji,J}g_{ij,J}}. & j,\, \ell \in J, j \neq \ell,\, g_{ji,J}g_{ij,J} \neq 1 \\ 0, & \text{otherwise} \end{cases}. \tag{13}$$

In general, the node-weights for any particular intersection hypothesis H_J, $J \subset I$ are uniquely defined by repeatedly applying (12-13) for all $i \in I\setminus J$. A corresponding algorithmic formulation of the procedure is given by Algorithm 1.

Algorithm 1 Compute weights for subset $J \subset I$

Input: G_I, $\boldsymbol{w}_I$
  $K \leftarrow I \setminus J$, $J' \leftarrow I$
  Let $(i_{(1)}, ..., i_{(k)})$ be any ordering of $i_{(j)} \in K$
  for $j = 1 \rightarrow k$ **do**
    Compute $\boldsymbol{w}_{J'\setminus i_{(j)}}$ according to (12)
    Compute $G_{J'\setminus i_{(j)}}$ according to (13)
    $J' \leftarrow J' \setminus i_{(j)}$
  end for
  $\boldsymbol{w}_J \leftarrow \boldsymbol{w}_{J'}$
  $G_J \leftarrow G_{J'}$

B Proof that the adaptive intersection hypothesis test controls the type I error rate

Under $H_J = \bigcap_{j\in J} H_j$, the marginal distribution of each p-value p_j, $j \in J$, is stochastically larger than or equal to the uniform distribution on the unit interval. Therefore, $E_{H_J}\left(\mathbf{1}_{\left\{p_j \le w_{j,J}\,\alpha\right\}}\right) \le w_{j,J}\,\alpha$. Consequently, taking the expectation of the partial conditional error function (2) over all $\mathcal{X}$ one has $E_{H_J}\left(A_{j,J}(w_{j,J}\,\alpha)\right) \le w_{j,J}\,\alpha$. Then for the sum of partial conditional error rates $B_J(\alpha)$ and an adapted test with decision function $\tilde{\varphi}_J$ that satisfies (4) it follows,

$$\begin{aligned} E_{H_J}\left(\tilde{\varphi}_J\right) = E_{H_J}\left[E_{H_J}(\tilde{\varphi}_J|\mathcal{X})\right] \le E_{H_J}\left[B_J(\alpha)\right] = \\ = \sum_{j\in J} E_{H_J}\left(A_{j,J}(w_{j,J}\,\alpha)\right) \le \sum_{j\in J} w_{j,J}\,\alpha \le \alpha, \end{aligned}$$

and consequently that the adaptive test controls the unconditional type I error rate for every intersection hypothesis test at local level α. Note that for a rigorous formulation of the procedure taking into account that the versions of the conditional expectations are in general not unique, one needs to assume that distribution of the second stage data is specified by a regression model in dependence of the interim data and the second-stage design [48].

C Computation of the case study with gMCP

The computation of the partial conditional error rates $A_{j,J}(w_{j,J}\,\alpha)$, the sums of partial conditional error rates B_J and the final test decision for the case study presented in Section 4 was performed with the following R-Code using an experimental part of the R-package gMCP Version 0.8-6 [50].

```
> library(gMCP)
>
> a <- .025 # one-sided significance level
> G <- simpleSuccessiveI() # First Stage Graph as defined in Figure 2
> ## some z-scores:
> z1 <- c(1.66,1.42,1.90,0.79) # first stage z-scores
> v <- c(1/2,1/2,1/2,1/2) # timing of interim analysis: n1/n
> intA <- doInterim(G,z1,v) # computation of partial conditional error rates
> intA # compare to Table 1
Pre-planned graphical MCP at level: 0.025
A graphMCP graph
H1 (weight=0.5)
H2 (weight=0.5)
H3 (weight=0)
H4 (weight=0)
Edges:
H1  -( 1 )->  H3
H2  -( 1 )->  H4
H3  -( 1 )->  H2
H4  -( 1 )->  H1

Proportion of pre-planned measurements
 collected up to interim:
 H1  H2  H3  H4
0.5 0.5 0.5 0.5
Z-scores computed at interim
  H1   H2   H3   H4
1.66 1.42 1.90 0.79

 Interim PCE's by intersection
           A(1)  A(2)  A(3)  A(4)    BJ
H(4)      0.000 0.000 0.000 0.024 0.024
H(3)      0.000 0.000 0.192 0.000 0.192
H(3,4)    0.000 0.000 0.102 0.009 0.111
H(2)      0.000 0.088 0.000 0.000 0.088
```

```
H(2,4)      0.000 0.088 0.000 0.000 0.088
H(2,3)      0.000 0.040 0.102 0.000 0.142
H(2,3,4)    0.000 0.040 0.102 0.000 0.142
H(1)        0.133 0.000 0.000 0.000 0.133
H(1,4)      0.066 0.000 0.000 0.009 0.074
H(1,3)      0.133 0.000 0.000 0.000 0.133
H(1,3,4)    0.066 0.000 0.000 0.009 0.074
H(1,2)      0.066 0.040 0.000 0.000 0.106
H(1,2,4)    0.066 0.040 0.000 0.000 0.106
H(1,2,3)    0.066 0.040 0.000 0.000 0.106
H(1,2,3,4) 0.066 0.040 0.000 0.000 0.106
>
> # Second Stage Graph as defined in Figure 3a
> m <- rbind(H1=c(0,0, 1,0 ),
+            H2=c(0, 0, 0, 0),
+            H3=c(0, 0, 0, 0),
+            H4=c(0, 0, 0, 0 )) # matrix of edge weights
> weights <- c(1, 0, 0, 0) # inital second stage weights
> graph <- new("graphMCP", m=m, weights=weights) # creation of graph
>
> z2 <- qnorm(1-c(.059,1,0.031,1)) # second stage z-scores
> selected=c(1,0,1,0) # selected hypotheses
> secondStageTest(intA,selected,G2=graph)(z2) # final test decisions
[1]  TRUE FALSE  TRUE FALSE
```
